# Exploring the Possibilities of Incorporating Edible Insects into a Vegetarian Diet: A Survey of Vegetarian Consumer Acceptance

**DOI:** 10.3390/nu16203572

**Published:** 2024-10-21

**Authors:** Ewelina Zielińska, Damian Zieliński

**Affiliations:** 1 Department of Analysis and Evaluation of Food Quality, University of Life Sciences in Lublin, Skromna Str. 8, 20-704 Lublin, Poland; 2 Department of Animal Ethology and Wildlife Management, University of Life Sciences in Lublin, Akademicka Str. 13, 20-950 Lublin, Poland; damian.zielinski@up.lublin.pl

**Keywords:** edible insects, entomophagy, survey, vegetarians

## Abstract

Background: Due to environmental, health, and ethical concerns, more consumers are reducing their meat consumption or giving it up entirely. Plant protein is most often chosen as a sustainable source of protein. Still, recently, edible insects have been gaining popularity as a source of alternative protein with a better nutritional profile. However, there is no information on whether vegetarians can accept insects. Methods: An online survey was conducted with a sample of 790 vegetarians to address this gap. The findings of this survey are crucial in understanding the potential acceptance of insects in vegetarian diets. Results: We found that 13% of the respondents approve of using processed insect protein in vegetarian dishes. Moreover, 9% of the respondents declared that they had knowingly consumed insects before; of these, 42% of them found the taste of the insects to be neutral, 16% found it to be very good, and 25% found it to be good. The level of insect acceptance was influenced by the type of vegetarian diet and its duration of use. Furthermore, pesca-vegetarians and flexi-vegetarians were the most likely to eat insects for ecological reasons ($\bar{x}$ = 3.54 ± 0.74; $\bar{x}$ = 3.00 ± 0.67, respectively). Conclusions: These findings do not eliminate the possibility of using edible insects in vegetarian diets but support their partial acceptance.

## 1. Introduction

Over the last few years, the range of alternatives to animal-based foods has steadily expanded, with plant-based food alternatives becoming the most popular. The search for alternatives to traditional foods is driven by various factors and one of the most important in recent few years is the environmental impact of the foods produced. As other motivations, consumers most often mention ethical (related to the welfare of farm animals) and health reasons.

The environmental impacts of food production and consumption are complex and include greenhouse gas emissions, land use for agriculture, and consumption of water resources. It is estimated that approximately 26% of human-caused greenhouse gas emissions stem from activities related to food production, processing, distribution, and consumption. Among these, agriculture-related emissions contribute to about 61% [1,2]. Livestock production is responsible for about 5% of anthropogenic CO_2_ emissions, 44% of CH_4_ emissions, and 53% of N_2_O emissions worldwide [2,3]. Another aspect of the environmental impact of food production is land use. According to the FAO, in 2019, agricultural activities occupied one-third of the world’s land area, mainly used for animal production with low protein conversion rates. In addition, livestock production consumes significant amounts of drinking water [2].

Plant-based substitutes are the most popular alternatives to animal-derived foods. The term ‘plant-based diet’ encompasses various dietary patterns that include smaller amounts of animal products and more significant amounts of plant-based products or exclude animal products entirely [4]. Plant-based alternative protein sources consistently have a lower overall environmental impact than their animal counterparts. The impact of alternative protein is 15 times lower than conventionally farmed beef and 5.5 times lower than chicken. However, even for eggs (the animal-based product with the lowest environmental footprint), the impact of the alternative was more than 3× lower than conventionally farmed eggs. By environmental impact, we mean greenhouse gas emissions, land use, and water consumption [5].

Moreover, the World Health Organisation recommends a diet containing 150–200 g of red meat and 500 g of white meat per week to stay healthy. Regrettably, the diets of numerous European residents greatly exceed these recommended amounts. The consumption of processed meat increases the risk of colorectal cancer. High amounts of saturated fatty acids and hem are considered the causative factor here. On the other hand, it is a fact that meat contains all the amino acids necessary for the synthesis of body proteins, enables the growth and development of the body, the reconstruction of cells, is important for defence processes, for the healing of wounds, and aids thought processes in the brain [6]. Therefore, it is important to compose a suitable alternative diet so that the body is supplied with all the essential amino acids.

A vegetarian is a person who does not eat meat, including poultry, seafood, fish, or products containing them. Vegetarianism has various subcategories, with some being less strict than others. Among the different types of vegetarian diets, there are ovo-lacto-vegetarians, who include dairy products and eggs in their diet; lacto-vegetarians, who include only milk; ovo-vegetarians, who include eggs; and the most restrictive vegans, who do not include any animal food at all [7]. There is also a growing number of flexitarians who consciously reduce their meat consumption or limit their intake to occasional consumption [8].

These three important issues, i.e., ecological, ethical, and health aspects, are also considered within the framework of insect consumption. The trend towards a healthy and balanced diet is present worldwide and entomophagy is part of this trend. The advantages of using insects include high feed conversion rates, low greenhouse gas emissions, low water consumption, and preserved animal welfare. Compared to traditional protein, insect protein is a good alternative because it has high nutritional value and digestibility with low environmental impact [9,10]. One of the insect species with the highest protein content is the house cricket (*Acheta domesticus*) (73.6%) [11]. Some insects are rich in fat, ranging from 4.5% to 60%. The fat content is higher in the larval stage than in the adult stage. Larvae of the greater wax moth (~60%) and mealworm (~43%) exhibit the highest amounts of fat [12]. Moreover, insects contain a wide range of bioactive components (e.g., chitin, polyphenols, antioxidant enzymes, antimicrobial peptides/proteins, etc.) that are responsible for their health-promoting potential [13]. Insect proteins and peptides represent various properties, including antimicrobial, antihypertensive, antioxidant, antidiabetic, hypocholesterolemic, anticancer, and hepatoprotective activity [14]. Due to the wide variety of edible insect species, we can identify several characteristics they possess. Most studies focused on insect protein-derived peptides with ACE inhibitory activity. Vercruysse et al. were among the first authors to present research on the ACE inhibitory activity of insect protein hydrolysates (*B. mori*, *B. terrestris*, *S. gregaria*, and *S. littoralis*) [15]. Peptides obtained from insects also have antibacterial and antifungal properties. A few antifungal compounds have been found in insects, for example, termicin from termites, drosomycin from *Drosophila melanogaster*, heliomicin from the tobacco budworm (*Heliothis virescens*), and the gallerimycin peptide from greater wax moth (*G. mellonella*) larvae. The extract from housefly larvae has a wide-ranging antibacterial effect against both Gram-negative and Gram-positive bacteria. *T. molitor* produces antimicrobial and antifungal tenecin 4 [16].

Combining insect food with a vegetarian diet is a very interesting issue. Insects, as invertebrates, are not as ethically controversial as vertebrates [17]. Moreover, they are more environmentally friendly, with an excellent nutritional profile and health-promoting properties. However, there is limited knowledge about the acceptance of insects in vegetarian diets. However, analyses were conducted on the acceptance of insects among people following a traditional diet, and a correlation was observed. Van Thielen et al. [18] indicated that a group of potential insect consumers eat less meat and do not do so daily. Kornher et al. [19] obtained consistent results. Their study shows that respondents who report infrequent and low consumption of meat products declare a higher probability of consuming insects in the future. In turn, the survey by Elorinne et al. [20] aimed to explore the attitudes and intentions of omnivores, non-vegan vegetarians, and vegans towards insect consumption. The authors concluded that non-vegan vegetarians had the most positive attitudes toward eating insects. Conversely, vegans had the most negative attitudes and expressed a low willingness to eat insects due to moral considerations. Observations show that insects can substitute meat in people’s diets, limiting consumption for vegetarians who have given up meat altogether. Most research has primarily focused on insect consumption by omnivores. However, it turns out that vegetarians and individuals limiting their meat intake are an important target group. In the literature, we can find the entoveganism concept. This is a niche dietary philosophy whose practitioners supplement a conventional plant-based diet with edible insects. This notion provides an important framework for considering insects as food and exploring the boundaries of morality, acceptability, edibility, and animalness. Moreover, it is argued that entoveganism decreases suffering on the following three levels: (1) the suffering of sentient animals involved in agro-industrial food systems, (2) the suffering of humans impacted by malnutrition and food insecurity, and (3) the future suffering of all life due to the effects of climate change [17]. These assumptions are important and certainly deserve wider consideration.

Our study aims to fill the knowledge gap by providing insight into the dietary habits of individuals following various vegetarian diets (veganism, lacto-vegetarianism, ovo-vegetarianism, lacto-ovo-vegetarianism, flexitarianism, and pesca-vegetarianism) and the acceptance of insects as part of the diet. In this study, a survey was conducted to explore the motivations of consumers following a vegetarian diet and their beliefs and intentions regarding the consumption of edible insects.

## 2. Materials and Methods

### 2.1. Data Collection and Respondent Profile

The experiment was based on a survey conducted on 790 vegetarians. Participants were recruited from an online access panel. The survey was distributed in internet forums and vegetarian discussion groups. The members of these groups voluntarily agreed to complete the survey and, following the link provided, they proceeded to complete it. We did not influence the number of people who volunteered to complete the questionnaire or the group’s structure in terms of metrics (gender, age, etc.), although they all declared that they followed a diet limited to animal products. All subjects gave informed consent for inclusion before participating in the study. This study was conducted in accordance with the Declaration of Helsinki (Brazil, 2013), and the protocol was approved by the Ethics Committee at the University of Life Sciences in Lublin (UKE/41/2024). Participation in the study was voluntary and was not associated with obtaining compensation.

### 2.2. Design of the Survey

The survey consisted of three parts: basic demographic questions, a section dedicated to the vegetarian diet, and a section exploring respondents’ views on edible insects (statements). The participants were asked about their sex, age, level of education, and place of residence. The vegetarian diet section addressed the duration of the diet and the motivations behind adopting it. In the final section, participants were questioned about the potential inclusion of insect protein in a vegetarian diet and their personal encounters with edible insects. The survey contained single-choice and multiple-choice questions and statements to which respondents were asked to respond (Table 1). All statements were presented using a Likert scale (1 = definitely not, 5 = definitely yes).

### 2.3. Statistical Analysis

Statistica (ver. 13,1, StatSoft Inc., Krakow, Poland) software was used for the statistical analysis. The validity of the collected data with a normal distribution was established using the Shapiro–Wilk test. As a result of confirming the conformity of the data with the normal distribution, the statistical analysis was performed using a one-way analysis of the variance (ANOVA) with the Tukey post hoc test when ANOVA results were statistically significant (*p* < 0.05 was a statistically significant difference). Statistical differences are shown in the results (on figures and tables) with different capital letters. The Chi-square test of independence (Χ^2^) was used to determine if there was a significant relationship between two categorical variables. Two-way ANOVA with interactions was used to calculate the interaction effect of two variables.

## 3. Results and Discussion

### 3.1. Respondents Profile

We asked 701 females (89%), 64 males (8%), and 25 genderqueers (3%) about their vegetarian diet and opinions about insect consumption. All participants were Polish. Most of them were aged 18–26 years old (70%) and lived in big cities (58%). Higher and secondary levels of education prevailed (49% and 47.5%, respectively). The most represented diet type was lact-ovo-vegetarianism and the largest group was respondents who had been on a diet for 3–5 years. The detailed respondents’ profile is presented in Table 2. We analysed survey results using various age ranges, place of residence, educational level, and job status to gain better insights. In the analysis, the vegetarian diet factors considered were the type of diet and the duration of adherence. The majority of participants followed a lacto-ovo-vegetarian diet for over a year.

### 3.2. Willingness to Eat Insects by Vegetarians

Of the 790 respondents following vegetarian diets, 13% approve of the use of processed insect protein in vegetarian dishes, while 16% could not specify their position. The type of diet used significantly influenced this opinion (Χ^2^ = 35.63; *df* = 10; *p* = 0.00010). Only 8% of vegans responded positively. Lacto-ovo-vegetarians were more open than lacto-vegetarians and ovo-vegetarians. Specifically, 14% of lacto-ovo-vegetarians approved of this possibility, compared to 9.5% for lacto-vegetarians and ovo-vegetarians. In turn, for flexitarians, it was 25%, and, for pesca-vegetarians, it was even 54%. The results aligned with our expectations. Pesca-vegetarians allow the consumption of fish and seafood in their diet; therefore, the consumption of insects also seems possible. Moreover, men were more favourable toward the idea (Χ^2^ = 15.24; *df* = 4; *p* = 0.00423). A total of 25% of men were in favour compared to 12% of women and 16% of genderqueer people.

In contrast, the length of time respondents have been on the diet showed no significance. Moreover, 9% (*N* = 70) of respondents declared that they had knowingly consumed insects before, and 42% of them found the taste of the insects to be neutral, 16% as very good, and 25% as good. An equal number of respondents specified it as bad and very bad (9%). Our previous study found that 15.5% of Polish respondents had tried insects, so the vegetarian group showed half as much interest in eating insects [21], whereas consumers’ evaluation of taste was very similar—17% of respondents identified the taste of the insects as very good, 40% as good, 21% as neutral and 19 of them were uncertain. Only 4% of participants found the taste bad [21]. However, the most up-to-date data on insect consumption are presented in the International Platform of Insects for Food and Feed (IPIFF) report. The survey sampled a total of 3000 people from Europe’s largest markets—Germany, France, Italy, Sweden, Poland, and Belgium—and, out of the total EU sample, 33% of the respondents had already eaten whole insects or food products made with insect ingredients. Moreover, in Poland, it was 39% [22]. This change is surprisingly significant. Appropriate information campaigns may also be possible to reach more vegetarians, such as pesca-vegetarians.

**Figure 1:**
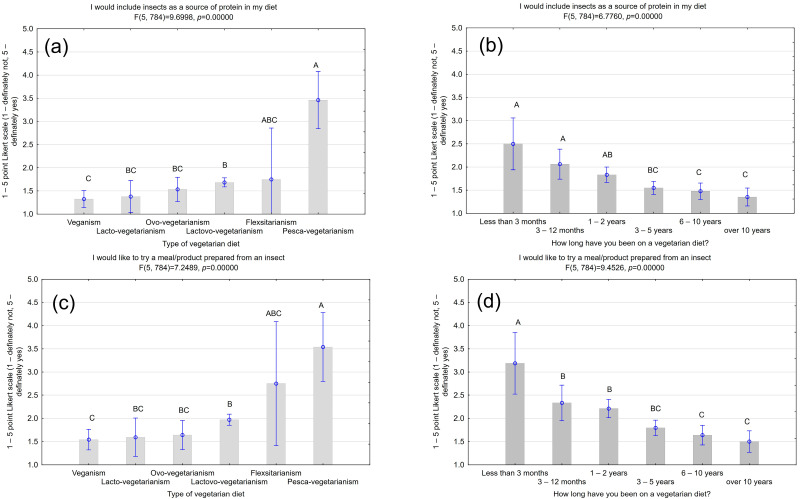
Willingness to eat insects. (**a**) Type of diet vs. willingness to include insects as a source of protein; (**b**) duration of the diet vs. the willingness to include insects as a source of protein; (**c**) type of diet vs. willingness to try a meal/product prepared from an insect; (**d**) duration of the diet vs. willingness to try a meal/product prepared from an insect. Vertical bars indicate 0.95 confidence intervals; ^A, B, C, ABC, BC, AB^ values with different capital letters differ significantly with *p* = 0.05 (Tukey’s test).

When responding to the question of whether respondents would be willing to include insects as a source of protein in their diet, statistically significant differences were shown between respondents declaring different types of vegetarian diets and the duration of their diet (F(5,784) = 9.6998, *p* = 0.00000; F(5,784) = 6.7760, *p* = 0.00000, respectively) (Figure 1). The pesca-vegetarians—vegetarians who also consume fish and shellfish—were the most interested in including insects in their diets ($\bar{x}$ = 3.46 ± 0.31). Understandably, then, because of the deviations present in their diets, they were more likely to declare such an interest than vegans or lacto-vegetarians ($\bar{x}$ = 1.33 ± 0.09; $\bar{x}$ = 1.38 ± 0.17, respectively), who were most pessimistic about such a possibility. As the diet duration increased, participants became increasingly against including insects. However, individuals with a short diet history (less than three months; $\bar{x}$ = 2.50 ± 0.28) were less committed compared to those who had been on the diet for more than ten years ($\bar{x}$ = 1.35 ± 0.09). A prolonged period of sticking to a diet can lead to stronger convictions, resulting in stricter adherence.

Pesca-vegetarians and flexitarians ($\bar{x}$ = 3.54 ± 0.38; $\bar{x}$ = 2.75 ± 0.68, respectively) were significantly more likely to consume dishes or products made from edible insects than respondents following other types of diets (Figure 1). Respondents were willing to try a dish or product containing insects in a similar pattern. People who had been on a diet for a short period were more likely to choose a meal containing insects ($\bar{x}$ = 3.19 ± 0.34) compared to those who had been on a diet for a more extended period ($\bar{x}$ = 1.50 ± 0.12)—the willingness to try a meal with insects decreased as the length of time on the diet increased (F(5,784) = 9.4526, *p* = 0.00000).

Gender had a strong influence on the answers to the above questions (Table 3). Males were more willing to include insects as a source of protein in their diet and to try a meal prepared from insects ($\bar{x}$ = 2.34 ± 1.60 and $\bar{x}$ = 2.55 ± 1.72, respectively). These results align with most studies, showing that gender is a significant predictor. Men are more likely than women to accept insects in various products, regardless of their visibility in food [23]. De Boer et al. suggest that men may be more inclined to take on challenges than women [24].

Additionally, age was a significant factor when considering insects as a source of protein in their diet (Table 3). As their age increases, respondents are more likely to include insects in their diet as a source of protein. This observation is interesting because, according to the theory of nutrition neophobia, disapproval of new types of food is more pronounced in the elderly than in young people [25]. However, a similar situation occurred in our previous study when we examined Polish consumers’ willingness to eat insects [21]. Moreover, Ros-Baró et al. [26] studied consumers’ acceptability of edible insects in Spain, and they stated that the most familiar with insect consumption was the 40–59-year-old age group. These results differ from the perception expressed by their respondents regarding greater acceptance by adolescents. The other two studies indicated that older individuals in Japan and China were more inclined to consume insects than younger ones due to their previous experience with insect consumption [27,28]. In many studies, younger individuals exhibited more positive attitudes toward insect-based foods than older individuals. However, other studies have also found that age did not significantly predict consumers’ acceptance of insect food [23].

In turn, the place of residence significantly influences the willingness to try a meal/product prepared from insects (Table 3). Residents of big cities were the most willing to consume this type of dish ($\bar{x}$ = 1.99 ± 1.45). Additionally, there were no significant differences in consumer attitudes based on education level and job status. Many studies agree with this conclusion [23]. Nevertheless, some studies suggest varying perceptions of insect consumption based on individuals’ education levels and residence places. For instance, a study conducted in Switzerland and Thailand by Brunner and Nuttavuthisit [29] revealed that the impact of education differed across cultures. In Switzerland, the early adopters of insects as food were more educated, whereas in Thailand, they were less educated. This was explained by the fact that highly educated people in Switzerland appeared to be more concerned about the sustainability and health aspects of entomophagy, while in Thailand, educated individuals associated entomophagy with Thailand’s rural traditions.

Furthermore, considering the ongoing acceptance of insects in Europe, we also inquired about the reasons that deter people from trying insects and which form of insects would be most convincing to consume. Representatives of all diet types stated that insects’ appearance is the most important factor discouraging their consumption. A total of 54% of lacto-ovo-vegetarians, 47% of vegans, 55% of ovo-vegetarians, 62% of pesca-vegetarians, and 50% of flexitarians chose this reason. We also inquired about which type of insect is most convincing for respondents to consume. A significant advantage among representatives of all kinds of vegetarian diets has been gained by the answer: the addition of protein isolated from insects to traditionally consumed products. Adding ground insects to traditionally consumed products was mentioned as the next most acceptable option.

Many studies indicate that consumers are more willing to eat invisible insects in familiar-looking and tasting food products than to accept a whole insect as food or the presence of unprocessed insects in a food product [30,31,32,33,34,35]. For this reason, research is being conducted to design recipes for various products that incorporate insects without negatively affecting their properties or consumer acceptance. These products are mainly bread [36,37,38,39,40], pasta [41,42], and snacks [43,44,45,46], which are readily and frequently consumed by consumers. However, Tan et al. [47] believe that even adequate product preparation is insufficient. Their research revealed that even when insects were prepared to resemble common foods, their perceived unsuitability as food had a negative impact on hedonic expectations and willingness to consume. The mealworm preparation, despite being invisible and having adequate flavour, was perceived as inferior to the original products, resulting in a low willingness to buy, even at the same price as the original products. At present, Western consumers generally do not consider insects as a regular part of their diet, even if they recognise their benefits and good taste. Therefore, to discuss consumer acceptance, it is crucial to prioritise education. This shift will lead to changed perceptions of insects and eliminate psychological barriers.

### 3.3. The Impact of the Ecological Nature of Insect Farming on Respondents’ Perceptions of It

We anticipated vegetarians to be most motivated by animal rights, ecology, and health concerns. As expected, respondents most frequently indicated these three aspects (multiple-choice questions), although considerations such as religious or economic factors were also raised. The reasons mentioned are relevant to our study because insect breeding significantly differs from livestock breeding. As invertebrates, insects are not as ethically contentious as vertebrates. They are also more environmentally sustainable and offer an excellent nutritional profile with numerous health benefits [14,48,49]. Thus, it seems to be a suitable option for at least some vegetarians. In a survey conducted by Sogari et al. [35] in 2018 and 2019 in Sydney, all vegetarians taking part in the survey (*N* = 18 and *N* = 23, respectively) cited their environmental concerns as a justification for choosing insect-based foods, especially compared to traditional meat production. They also recognised this as a way that could be used to address the present and impending climate change on our planet.

**Figure 2:**
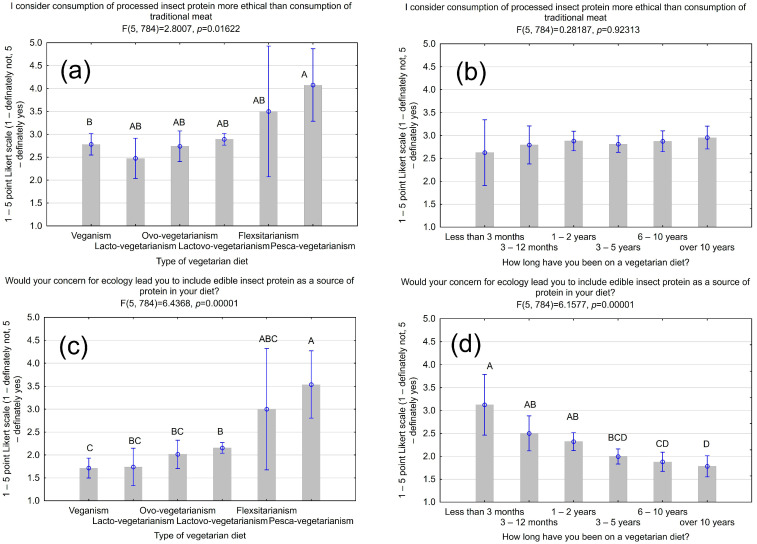
The impact of the ecological nature of insect farming on respondents’ perceptions of it. (**a**) The belief that the consumption of processed insect protein is more ethical than the consumption of traditional meat vs. the type of diet used; (**b**) the belief that the consumption of processed insect protein is more ethical than the consumption of traditional meat vs. the duration of the diet; (**c**) finding that ecological concerns would prompt the inclusion of edible insects as a source of protein vs. the type of diet used; (**d**) finding that ecological concerns would prompt the inclusion of edible insects as a source of protein vs. the duration of the diet. Vertical bars indicate 0.95 confidence intervals; ^A, B, C, D, AB, BC, ABC, BCD, CD^ values with different capital letters differ significantly with *p* = 0.05 (Tukey’s test).

We asked respondents whether they thought it was more ethical to consume processed insect protein than traditional meat. The type of diet significantly impacted the results (F(5,784) = 2.8007, *p* = 0.01622) (Figure 2). Pesca-vegetarians ($\bar{x}$ = 4.08 ± 0.35) and flexitarians ($\bar{x}$ = 3.5 ± 0.96) were the most supportive of this statement. This finding aligns with the fact that these vegetarian groups are willing to consume insects, possibly because they consider eating insects more ethical than traditional meat. In a survey of Dutch consumers, it was found that vegetarians may choose to eat insect-based foods for environmental reasons as well as because insects are perceived as lacking sensitivity or the capacity to suffer, which is considered more ethical than eating livestock [50].

**Figure 3:**
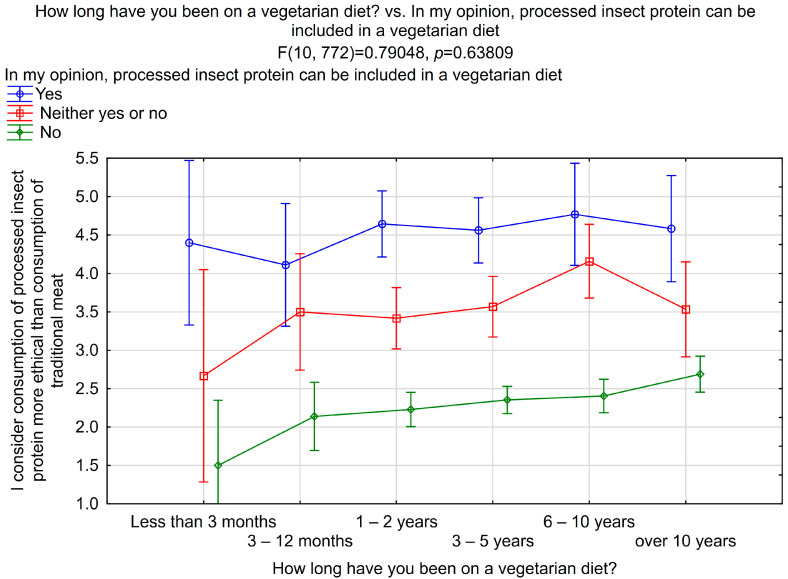
The interaction between the duration of the diet, the willingness to include insects in a vegetarian diet, and the perception that the consumption of processed insect protein is more ethical than the consumption of traditional meat. Vertical bars indicate 0.95 confidence intervals.

Gender, age, and place of residence strongly influenced the answers to this question (Table 3). Generally, males and genderqueer individuals ($\bar{x}$ = 3.66 ± 1.52 and $\bar{x}$ = 3.24 ± 1.48, respectively), respondents over 36 years old ($\bar{x}$ = 3.35 ± 1.53), and residents of big cities ($\bar{x}$ = 3.04 ± 1.46) were more convinced that consuming processed insect protein is more ethical than consuming traditional meat. This is consistent with previously discussed questions where the same groups were more likely to introduce insects into their diet. Furthermore, respondents who declared that processed insect protein could be included in a vegetarian diet consider the consumption of processed insect protein more ethical than consuming traditional meat (Figure 3). As the duration of a vegetarian diet increased, so did the belief that consuming processed insect protein was more ethical than consuming traditional meat among those opposed to including insects in a vegetarian diet.

Respondents were also asked about their attitudes toward ecology (statement: ecology matters significantly to me). Overall, all the participants believed that ecology was important to them, and the type of diet they followed significantly influenced their scores (F(5,784) = 2.9494, *p* = 0.01203). The highest scores were achieved by vegetarians ($\bar{x}$ = 4.59 ± 0.06), while the lowest scores were achieved by pesca-vegetarians ($\bar{x}$ = 3.92 ± 0.21). The duration of the diet did not significantly differentiate the results.

Later in the survey, we clarified that insects are considered a more environmentally friendly source of protein than livestock due to lower greenhouse gas emissions and reduced drinking water and feed consumption. Respondents were asked whether their ecological approach influenced their willingness to include edible insects as a protein source in their diet. Both the type of diet used and the duration of the diet had a significant impact on the results (F(5,784) = 6.4368, *p* = 0.00001; F(5,784) = 6.15.77, *p* = 0.00001) (Figure 2). Vegans ($\bar{x}$ = 1.17 ± 0.11) were the most critical of the question asked, while pesca-vegetarians and flexitarians ($\bar{x}$ = 3.54 ± 0.74; $\bar{x}$ = 3.00 ± 0.67, respectively) were the most likely to eat insects for ecological reasons. The willingness to include edible insects as a source of protein in the diet decreased with the duration of the diet. Moreover, male and genderqueer individuals ($\bar{x}$ = 2.64 ± 1.59; $\bar{x}$ = 2.68 ± 1.49, respectively) and respondents over 36 years old ($\bar{x}$ = 2.44 ± 1.58) were more accepting of this statement than women ($\bar{x}$ = 1.99 ± 1.33) and younger people ($\bar{x}$ = 2.03 ± 1.33 for 18–26 years old and $\bar{x}$ = 1.93 ± 1.36 for 27–35 years old) (Table 2).

In the Sogari et al. [35] study, respondents identified three main environmental factors that encourage the choice of insects as a meat alternative: less greenhouse gas emissions, sustainable farming of insects, and a better environment for the planet. Moreover, Ecology, environmental considerations, and ensuring a more sustainable future were commonly cited by omnivorous and non-vegan vegetarians as reasons for consuming insect food, as indicated in the Elorinne et al. [20] study.

## 4. Conclusions

Western countries do not have a tradition of consuming insects, but numerous studies investigate their acceptance among the public. Their increasing market presence requires a thorough analysis of the potential for European consumers to start eating insects soon. Our study was designed to investigate the acceptance of insects among individuals following a vegetarian diet, a trend gaining popularity. Our study suggests that vegetarians accept the consumption of insects to a lesser extent than omnivores but do not exclude it altogether. These results could guide insect food manufacturers in developing a product line for vegetarians based on a detailed analysis of their preferences.

A total of 13% of respondents approve of using processed insect protein in vegetarian dishes, mainly in insect protein isolates or insect powder. Moreover, the type of diet used significantly influenced this opinion. The most supportive of the use of insects in a vegetarian diet were pesca-vegetarians, who were surprisingly positive about this possibility, with as many as 54% in favour. Ethical and ecological issues related to insect consumption were also considered, and attitudes towards insects varied based on the type and duration of the diet. Generally, vegetarians are an important group who can accept insects in their diet, but appropriate information and education campaigns could influence their attitudes.

We also believe further research is necessary to thoroughly analyse the attitudes of the increasing number of vegetarians. Further research could focus on a more detailed examination of the attitudes of vegetarians towards specific insect species. Even among those who declared an interest in consuming insects, there may be preferences in the species selection and the form in which the insects are consumed. There are over 2000 edible insect species, each with different nutritional profiles and welfare needs [51]. Further research specific to each insect species is necessary to define their welfare needs and confirm consumer acceptance. Another important aspect is examining the physiological effects of vegetarians’ insect consumption. This type of study would likely involve an in-depth analysis of the utilisation of insects in a vegetarian diet, considering the impact of their consumption on the body.

## Figures and Tables

**Table 1:** Design of the survey.

	Question	Answers	Type of Response
Description of the respondent	Sex	Female/Male/Genderqueer	Single choice
	Age	18–26 years old/27–35 years old/over 36 years old	Single choice
	Place of residence	Rural area/Small city/Big city	Single choice
	Educational level	Primary/Secondary/Big city	Single choice
	Job status	Student/Unemployed/Working/Retiree	Single choice
Questions about vegetarian diet	Type of vegetarian diet	Lact-ovo-vegetarianism/Veganism/Lacto-vegetarianism/Ovo-vegetariansm/Pesca-vegetarianism/Flexitarianism	Single choice
	Length of time on the diet	Less than 3 months/3–12 months/1–2 years/3–5 years/6–10 years/over 10 years	Single choice
	Reasons for adopting a vegetarian diet	Ethical/Environmental/HealthEconomic/Religious/I don’t like meat/My family uses it/Other	Multiple choice
	Ecology matters significantly to me	Checkbox: 1 2 3 4 5	5-point Likert scale
Questions about insects in vegetarian diet	Have you ever tried eating insects?	Yes/No	Single choice
	If yes, how would you rate their taste?	Very good/Good/Neutral/Bad/Very bad	Single choice
	What discourages you the most from trying insects?	Appearance/Images of bad taste/Images of bad smell/There is nothing like that	Single choice
	Which form of insect consumption do you find most convincing?	Eating insects whole as a dish/Addition of whole insects to traditionally consumed products/Addition of ground insects to traditionally consumed products/Addition of protein isolated from insects to traditionally consumed products	Single choice
	In my opinion, processed insect protein can be included in a vegetarian diet.	Yes/No/I have no opinion	Single choice
	I would include insects as a source of protein in my diet.	Checkbox: 1 2 3 4 5	5-point Likert scale
	I would like to try a meal/product prepared from an insect.	Checkbox: 1 2 3 4 5	5-point Likert scale
	I consider the consumption of processed insect protein more ethical than the consumption of traditional meat.	Checkbox: 1 2 3 4 5	5-point Likert scale
	Insects are considered a more environmentally friendly protein source than livestock, producing fewer greenhouse gas emissions and requiring less drinking water and feed. Would your concern for ecology lead you to include edible insect protein as a source of protein in your diet?	Checkbox: 1 2 3 4 5	5-point Likert scale

**Table 2:** Socio-demographic characteristics of respondents (*N*, %).

		*N*	%
Sex	Female	701	89
	Male	64	8
	Genderqueer	25	3
Age	18–26 year old	553	70
	27–35 years old	135	17
	Over 36 years old	102	13
Place of residence	Rural area	132	17
	Small city	200	25
	Big city	458	58
Educational level	Primary	28	3.5
	Secondary	375	47.5
	Higher	387	49
Job status	Student	401	50.7
	Unemployed	33	4.2
	Working	355	45
	Retiree	1	0.1
Type of vegetarian diet	Lact-ovo-vegetarianism	511	64.7
	Veganism	147	18.6
	Lacto-vegetarianism	42	5.3
	Ovo-vegetarianism	73	9.2
	Pesca-vegetarianism	13	1.7
	Flexitarianism	4	0.5
Length of time on the diet	Less than 3 months	16	2
	3–12 months	48	6
	1–2 years	181	23
	3–5 years	253	32
	6–10 years	159	20
	Over 10 years	133	17

**Table 3:** Willingness to accept insects according to gender, age, and place of residence.

		Statement (5-Point Likert Scale: 1—Definitely Not, 5—Definitely Yes)											
		I would include insects as a source of protein in my diet.			I would like to try a meal/product prepared from an insect.			I consider the consumption of processed insect protein more ethical than the consumption of traditional meat.			Would your concern for ecology lead you to include edible insect protein as a source of protein in your diet?		
		Mean (sd)	F-value	*p*	Mean (sd)	F-value	*p*	Mean (sd)	F-value	*p*	Mean (sd)	F-value	*p*
Gender	Female	1.55 ^B^ ± 1.09	14.25	0.000001	1.80 ^B^ ± 1.33	9.04	0.0001	2.77 ^B^ ± 1.43	11.95	0.00008	1.99 ^B^ ± 1.33	9.38	0.00009
	Male	2.34 ^A^ ± 1.60			2.55 ^A^ ± 1.72			3.66 ^A^ ± 1.52			2.64 ^A^ ± 1.59		
	Genderqueer	1.68 ^B^ ± 1.25			2.12 ^AB^ ± 1.54			3.24 ^A^ ± 1.48			2.68 ^A^ ± 1.49		
Age	18–26 years old	1.55 ^B^ ± 1.07	5.37	0.005	1.82 ± 1.34	2.51	0.08	2.77 ^B^ ± 1.43	6.92	0.001	2.03 ^B^ ± 1.33	4.71	0.009
	27–35 years old	1.62 ^B^ ± 1.20			1.36 ± 1.39			2.84 ^B^ ± 1.46			1.93 ^B^ ± 1.36		
	Over 36 years old	1.96 ^A^ ± 1.48			1.58 ± 1.61			3.35 ^A^ ± 1.53			2.44 ^A^ ± 1.58		
Place of residence	Rural area	1.49 ± 1.03	2.68	0.07	1.80 ^AB^ ± 1.37	4.14	0.01	2.67 ^B^ ± 1.48	9.23	0.0001	1.99 ± 1.33	1.92	0.15
	Small city	1.52 ± 1.07			1.66 ^B^ ± 1.21			2.56 ^B^ ± 1.38			1.93 ± 1.28		
	Big city	1.70 ± 1.23			1.99 ^A^ ± 1.45			3.04 ^A^ ± 1.46			2.14 ± 1.42		

^A, B, AB^ values with different capital letters differ significantly with *p* = 0.05 (Tukey’s test).

## Data Availability

The data presented in this study are available on request from the corresponding author.
